# APOE4 dysregulates autophagy in cultured cells

**DOI:** 10.1080/27694127.2022.2040767

**Published:** 2022-03-22

**Authors:** Gianna M. Fote, Joan S. Steffan

**Affiliations:** a Department of Biological Chemistry, University of California, Irvine, Irvine, California, USA;; b Department of Psychiatry and Human Behavior, and UCI MIND Institute, University of California, Irvine, Irvine, California, USA

**Keywords:** Alzheimer’s disease, APOE, APOE4, chaperone-mediated autophagy, LAMP2A

## Abstract

Human APOE4 (apolipoprotein E4 isoform) is a powerful genetic risk factor for late-onset Alzheimer disease (AD). Many groups have investigated the effect of APOE4 on the degradation of amyloid β (Aβ), the main component of plaques found in the brains of AD patients. However, few studies have focused on the degradation of APOE itself. We investigated the lysosomal trafficking of APOE in cells and found that APOE from the post-Golgi compartment is degraded through an autophagic process requiring the lysosomal membrane protein LAMP2A. We found that APOE4 accumulates in enlarged lysosomes, alters autophagic flux, and changes the proteomic contents of lysosomes following internalization. This dysregulated lysosomal trafficking may represent one of the mechanisms that contributes to AD pathogenesis.

Late-onset AD is the most common form of dementia, with one in ten Americans over the age of 65 affected. There are 3 human *APOE* alleles (*APOE2, APOE3* and *APOE4*) encoding proteins differing by only one or two amino acids. A single copy of *APOE4* confers a threefold greater risk of developing AD, while two copies confer a fifteen-fold greater risk. APOE4 protein levels are lower than APOE3 in human serum, plasma, brain, and cultured cells. Proposed novel therapeutic strategies for AD include both increasing APOE function (APOE mimetics, increasing APOE4 lipidation, small molecule APOE4 structure correctors, *APOE2* gene therapy) and reducing APOE expression using antisense oligonucleotides. Whereas these novel therapeutic strategies seek to modulate abundance and function of APOE4, endogenous mechanisms of APOE trafficking and degradation are incompletely understood. In order to track the flow of APOE through the endolysosomal system, we developed novel cell lines expressing fluorescently tagged APOE3 and APOE4 [1]. Consistent with previous studies showing that expression of APOE4 damages lysosomal membrane integrity, dysregulates endo/lysosomal pH, and results in enlarged endosomes in various models, we found that APOE4 accumulates in enlarged lysosomes and late endosomes (Figure 1). APOE levels increase with lysosomal inhibitors even in the presence of endocytosis inhibitors, suggesting that in these cell culture systems APOE is degraded intracellularly by autophagy.

We further interrogated autophagic mechanisms and found that degradation of APOE requires trafficking out of the Golgi apparatus, and that knockdown of the chaperone-mediated autophagy (CMA) receptor protein LAMP2A inhibits APOE degradation in multiple cell lines. These results are in agreement with a recent study of *lamp2a* KO mouse models that found accumulation of APOE in brain tissue. CMA has historically been defined as only degrading cytoplasmic proteins. It is therefore surprising that CMA may contribute to degradation of APOE, which is presumably contained within secretory vesicles. One possible explanation is that LAMP2A complexes at the lysosomal surface may contribute to invagination of the lysosomal membrane around unconventional CMA substrates through a microautophagy-like process (Figure 2A). This may explain degradation of APOE-containing vesicles and previous reports of degradation of Aβ oligomers by CMA. An alternative explanation for the degradation of APOE by CMA could be release of APOE from the secretory vesicular system (Figure 2B). There is some literature showing that APOE can escape into the cytoplasm in neurons and hepatocytes. APOE has been observed outside the secretory system in the nucleus, mitochondria, and mitochondria-derived vesicles.

Having observed that APOE4 accumulates in lysosomes, we investigated whether this accumulation may perturb autophagic flux and the degradation of various autophagic substrates. Similar to recent studies showing that APOE4 downregulates autophagy, we find higher levels of LC3-II in APOE4-expressing cells; this effect is not further modulated by bafilomycin A_1_ treatment, suggesting that there might be a late-stage block in autophagic flux at the level of the lysosome. However, increased BECN1 levels and LC3 abundance in APOE4 cells suggest that this block may be exacerbated by activation of macroautophagy at the initiation stage. Degradation of lipid droplets by lipophagy is enhanced in these APOE4-expressing cells, possibly in order to supply the necessary lipids to form autophagosomes. The combination of enhanced autophagy initiation with a late-stage block may contribute to the accumulation of autophagosomes that has been observed in AD brain tissue and could be quite cytotoxic. To further investigate how APOE4 may dysregulate autophagy, we performed proteomics analysis of lysosomes isolated from cells endocytosing APOE4, and found reduced levels of mitochondrial proteins, consistent with previous reports that APOE4 perturbs mitophagy.

In a number of proteopathic neurodegenerative diseases, mutant proteins can contribute to disease through both gain and loss of normal protein function. One possibility that cannot be ruled out is that in addition to being aberrantly trafficked through the lysosomal system, APOE4 may perturb autophagy by failing to perform functions fulfilled by APOE3. Autophagy proteins are typically cytoplasmic rather than lipid-bound proteins contained within secretory vesicles, but if APOE escapes into the cytoplasm to be degraded by CMA, it may itself also be a part of the autophagic machinery. It remains to be seen in *apoe* knockout systems whether any autophagic substrates require APOE for trafficking to lysosomes.

In summary, our results suggest that APOE is degraded by LAMP2A-dependent autophagy, and that APOE4 accumulates in dysfunctional lysosomes. Future clinically translational studies could investigate whether increased lysosomal trafficking of APOE4 is protective or harmful, and whether autophagy can be manipulated to improve trafficking of APOE4 and other autophagic substrates in AD. Modulation of different types of autophagy, such as CMA and macroautophagy, and further investigation of the autophagic balance throughout the course of disease, may better inform future therapeutic strategies.

## Figures and Tables

**Figure 1.**
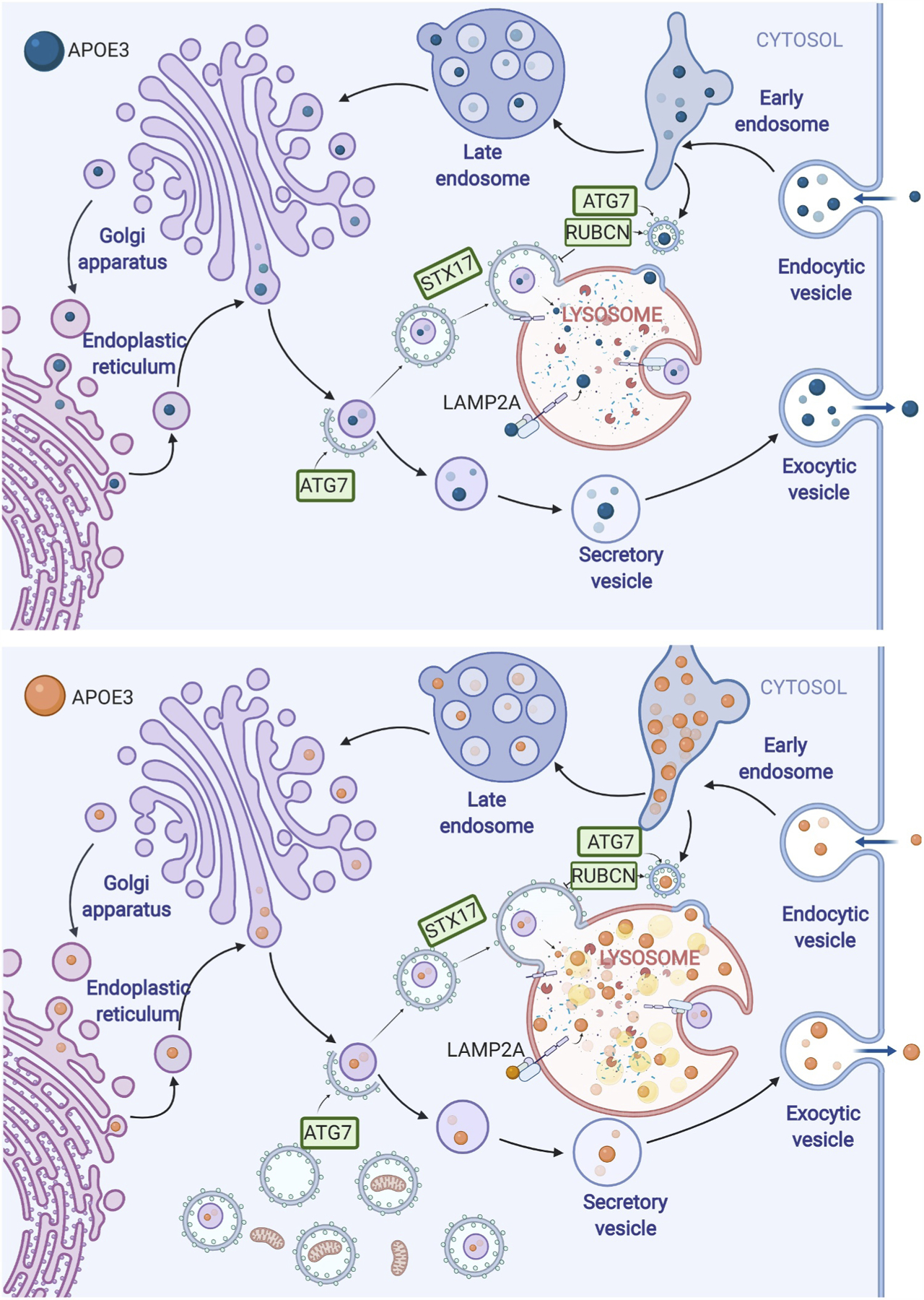
Aberrant lysosomal trafficking of APOE4 (below, orange) vs. APOE3 (above, blue). APOE is trafficked to the lysosome from the post-Golgi compartment, and accumulates in late endosomes and lysosomes. APOE4 perturbs autophagic flux, causes an accumulation of LC3-II and prevents mitochondrial proteins from reaching the lysosome.

**Figure 2.**
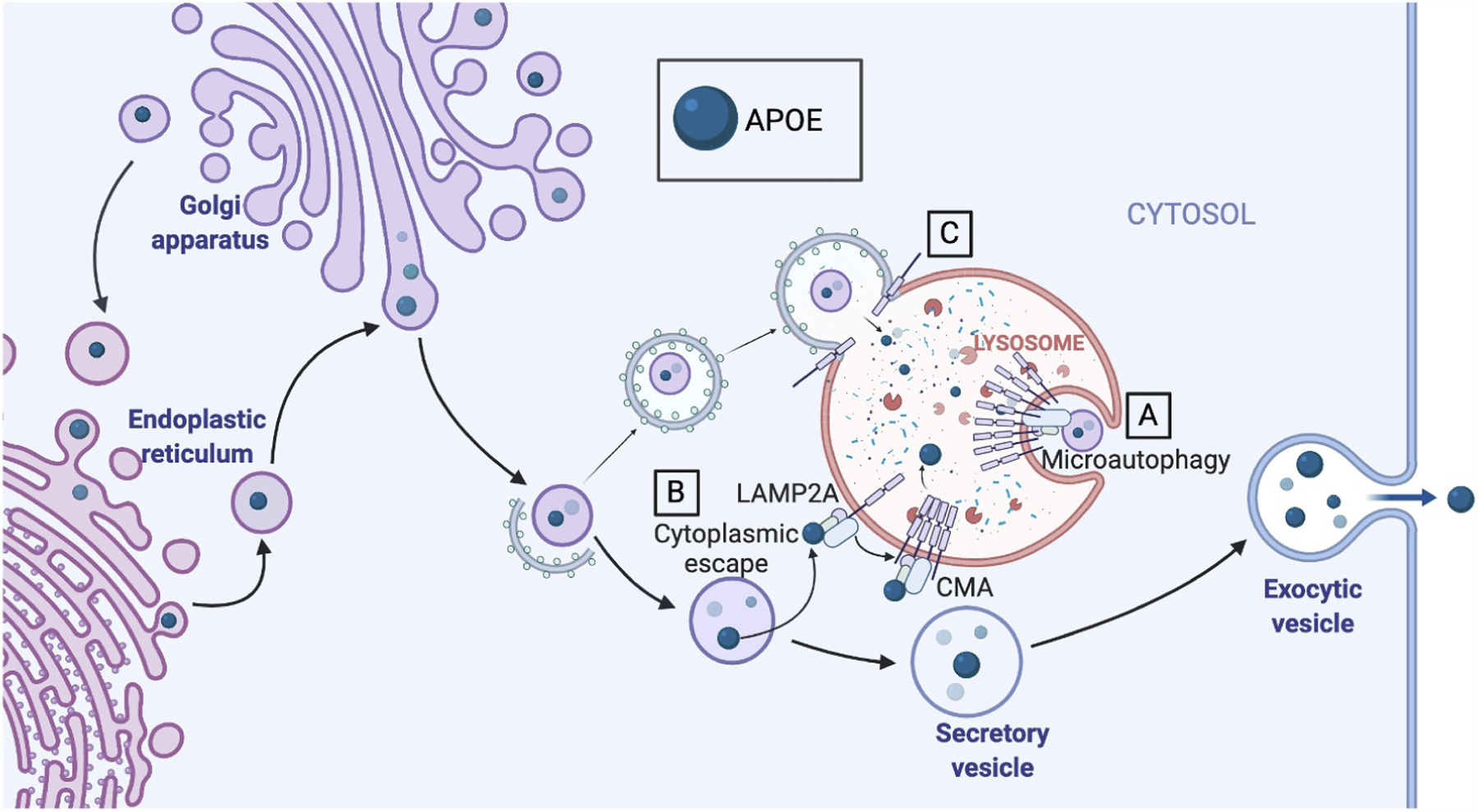
APOE is degraded by LAMP2A-dependent autophagy. (A) LAMP2A may participate in microautophagy of secretory vesicles containing APOE. (B) APOE may escape secretory vesicles into the cytoplasm, where it can be recognized by HSPA8/HSC70, which then binds to LAMP2A. LAMP2A forms a multimer on the lysosomal surface and facilitates the transfer of APOE into the lysosomal lumen by CMA. (C) LAMP2A also functions in autophagosomal fusion. APOE is degraded by macroautophagy; however, knockdown of LAMP2A has an additive effect with knockdown of other macroautophagy proteins, making it more likely that multiple separate autophagic processes are contributing to degradation of APOE.
